# One-Year Review of Novel “Virtual” Pneumonia Follow-Up Clinic a Decade From Inception

**DOI:** 10.7759/cureus.85892

**Published:** 2025-06-12

**Authors:** Malin White, Mian W Ahmed, Emma Lyons, Ffion Elliott, Ritwick Chatterji, Yasmin Choudhury, Arun Joshi, Ben Marshall

**Affiliations:** 1 Pulmonary Medicine, Southampton General Hospital NHS Foundation Trust, Southampton, GBR; 2 Respiratory Medicine, Southampton General Hospital NHS Foundation Trust, Southampton, GBR

**Keywords:** cost-effectiveness, pneumonia follow-up, respiratory medicine, service evaluation, virtual clinic

## Abstract

Background

Follow-up after pneumonia is essential to detect underlying malignancies and identify residual complications. In 2013, the University Hospitals of Southampton NHS Foundation Trust implemented a novel virtual pneumonia clinic (VPC) to reduce outpatient burden while maintaining clinical standards by providing remote consultant-led follow-up for patients with pneumonia. This study reviews the service a decade after its inception.

Methods

We conducted a retrospective analysis of all referrals to the VPC between January 2022 and January 2023. Data on demographics, referral source, imaging outcomes, time to follow-up, and final diagnoses were collected. Patients were stratified by age and smoking status, and outcomes such as attendance, residual abnormalities, and diagnostic escalation were analysed.

Results

A total of 891 patients were referred during the 12 months, representing a threefold increase since the service’s inception. Overall, 78.0% attended their convalescent chest X-ray (CXR), with the highest yield of abnormalities seen in patients over 80. Residual radiographic abnormalities were identified in 6.6% of attendees, including lung cancer, pneumonia-related scarring, pleural effusion, and bronchiectasis. The mean time to follow-up CXR was 51.0 days (interquartile range (IQR): 35-63). Referrals were predominantly from respiratory and emergency teams. Based on these findings, referral criteria have been refined to target patients over 50 or those with persistent symptoms or respiratory risk factors.

Discussion

The VPC model has demonstrated both clinical efficacy and operational efficiency. Consultant time was reduced by 92%, with an estimated per-patient cost saving of £200. Broader NHS and international evidence support virtual models in reducing hospital utilisation, environmental impact, and increasing patient satisfaction.

Results

The VPC continues to provide a scalable, sustainable model of care, combining diagnostic vigilance with outpatient efficiency. Our findings support replication across other NHS trusts and healthcare systems seeking to enhance post-discharge follow-up while managing resources effectively.

## Introduction

We have conducted a service review of the most recent 12 months of our virtual pneumonia follow-up clinic (VPC), marking the 10-year anniversary since its inception.

Pneumonia is a common and often serious condition, with an estimated 35-70,000 cases annually in the UK. It accounts for 5-12% of lower respiratory tract infections seen in general practice, contributing significantly to hospital admissions and NHS resource use [1]. Effective treatment and timely follow-up are essential to ensure clinical recovery, identify complications such as persistent radiographic abnormalities or malignancy, and support safe discharge planning.

The importance of follow-up for patients with community-acquired pneumonia has long been recognised. Seminal studies by Woodhead (1987) and Macfarlane (1982) informed current British Thoracic Society (BTS) guidelines, which recommend repeat chest imaging after six weeks for patients over 50, those with ongoing symptoms, or those at high risk of underlying pathology [2-4].

University Hospitals of Southampton (UHS) NHS Foundation Trust is a tertiary centre and teaching hospital serving a local population of 500,000 people and a regional population of 2 million. In 2014, the UHS Trust established a novel VPC as a consultant-led service designed to streamline follow-up care. The aim was to reduce unnecessary outpatient visits while ensuring early identification of clinically important residual findings. Over the past decade, this virtual model has grown steadily in demand, with over 300% growth in referrals. This study presents a 12-month review of the VPC a decade after its inception.

Study objectives

This study aimed to evaluate the VPC by assessing referral volumes, attendance rates, radiographic outcomes, diagnostic yield, timeliness of follow-up, and operational efficiency. Additionally, we report recent refinements to the referral criteria and discuss broader implications for NHS outpatient transformation and sustainable healthcare delivery.

This article was previously delivered as an oral poster presentation with a published abstract at the 2024 BTS Annual Winter Meeting on November 27, 2024.

## Materials and methods

We analysed data from January 2022 to January 2023, taking advantage of a dedicated database set up for usage in the VPC. A total of 891 patients were referred to the VPC during this period, and data were collected retrospectively. This was nearly three times the number referred in the first year of the service in 2013 [5].

For each patient referred between January 2022 and January 2023, we extracted demographic information (age, gender, smoking status), referral source (e.g., Emergency Department, Respiratory or Medical teams), and clinical details including date of pneumonia diagnosis, date of initial and convalescent chest X-rays, and attendance status. Radiological findings from follow-up imaging were recorded, including any persistent abnormalities such as scarring, pleural effusion, bronchiectasis, or suspected malignancy. Where applicable, outcomes from further investigations (e.g., CT scans, multidisciplinary team (MDT) discussions) were also documented. Additional metrics captured included time intervals between referral and convalescent imaging, reasons for non-attendance, and mortality prior to follow-up. This comprehensive dataset allowed for stratified analysis by age group and evaluation of diagnostic yield, service efficiency, and clinical impact.

Clinicians can make a referral to this service via an electronic referral process. A letter is then sent to the patient with an invitation to attend for a convalescent X-ray, which is booked in a centre convenient for the patient to attend. The virtual clinic is run by a consultant respiratory physician and a specialist nurse, two to four times a month, to review the X-rays of patients who have attended for a convalescent X-ray. Template letters are sent out to the patients, and a copy is sent to the general practitioner (GP), giving the results of the final X-ray and instructions for any further investigations required. Suspected malignancies were confirmed through CT imaging, bronchoscopy, and/or histological evaluation following referral to a two-week wait (2WW) lung cancer pathway and discussed at the respiratory MDT. All radiographic reviews were conducted by a consultant respiratory physician and discussed with a consultant radiologist where necessary.

We focused on attendance rates and residual positive findings. If the patient failed to attend on the first invitation, a second and final invitation letter was sent. If a second default occurred, the patient was discharged from the service with a safety-netting letter issued to the patient’s GP.

**Figure 1:**
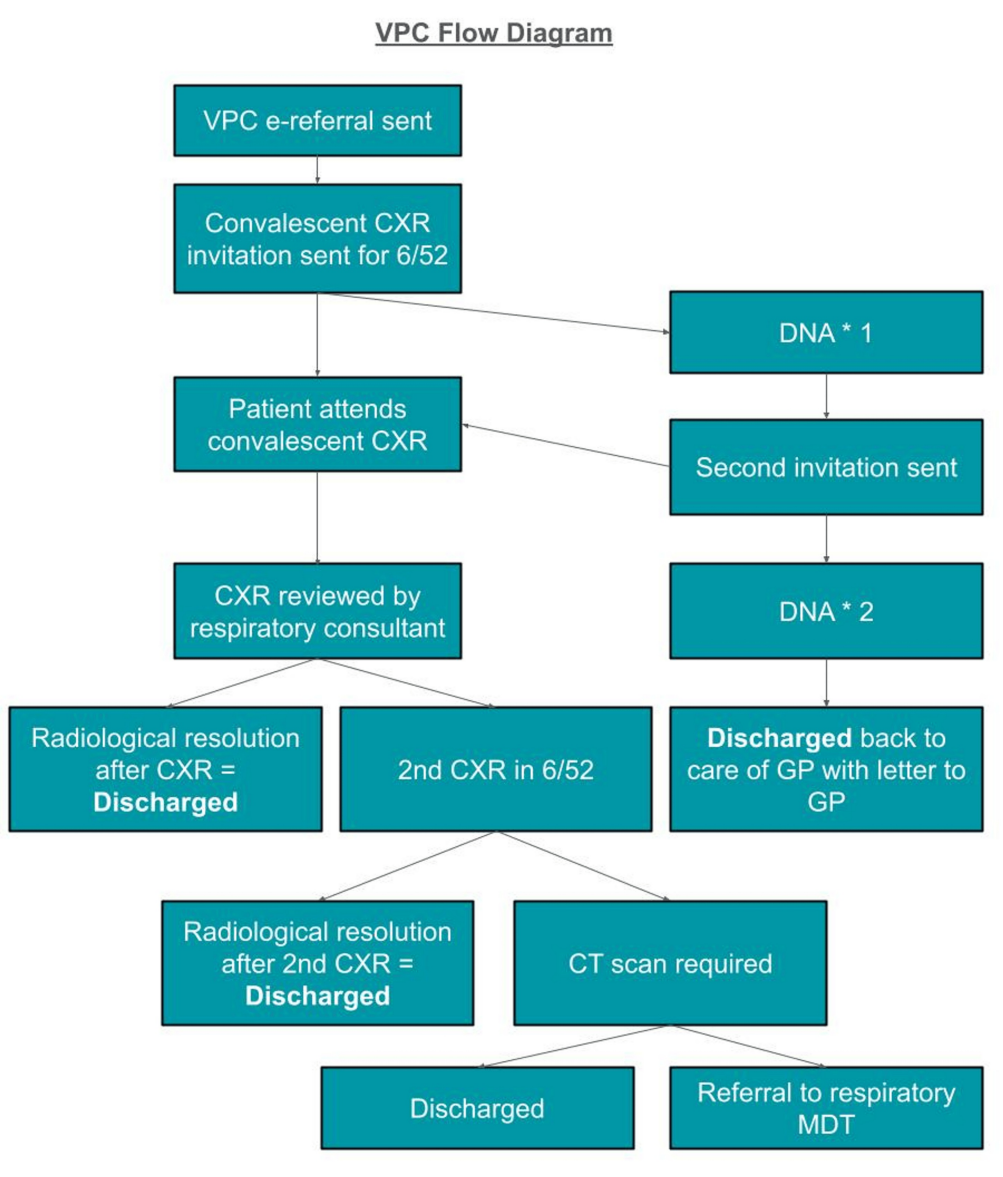
Flow diagram of the virtual pneumonia clinic (VPC) pathway. Patients referred electronically receive an invitation for a convalescent chest X-ray (CXR) six weeks post-discharge. If they do not attend (DNA), a second invitation is sent. Persistent non-attendance results in a general practitioner (GP) notification. CXRs are reviewed virtually by a respiratory consultant. Depending on radiographic findings, patients are either discharged, invited for a second CXR, or referred for CT imaging and multidisciplinary team (MDT) review.

A visual representation of this process is provided in Figure 1.

Data was analysed descriptively using Microsoft Excel. Categorical variables were reported as counts and percentages, and continuous variables (e.g., time to imaging) as a mean and interquartile range (IQR).

This project was registered as a service evaluation with the UHS NHS Foundation Trust and did not require formal ethical approval.

## Results

A total of 891 patients were referred to the VPC between January 2022 and January 2023. Referrals were stratified into three age groups: <40 years (n=105, 11.8%), 40-80 years (n=565, 63.3%), and >80 years (n=221, 24.8%) (Table 1).

**Table 1:** Virtual Pneumonia Clinic Referrals by Age Group, Attendance, and Radiological Findings This table presents the total number of referrals, attendance at convalescent chest X-ray (CXR), and number of residual abnormalities detected, stratified by age group (<40, 40–80, and >80 years).

Age	Total Referrals	Attended Convalescent X-ray	Residual Abnormality
>80	221	164	17
40-80	565	445	27
<40	105	79	2

Attendance rates

Overall, 695 of 891 patients (78.0%) completed a convalescent CXR. Attendance rates were 75.2% (n=79/105) in the <40 group, 78.9% (n=445/564) in the 40-80 group, and 74.2% (n=164/221) in the >80 group. Patients who did not attend after two invitations were discharged with a safety-netting letter.

**Figure 2:**
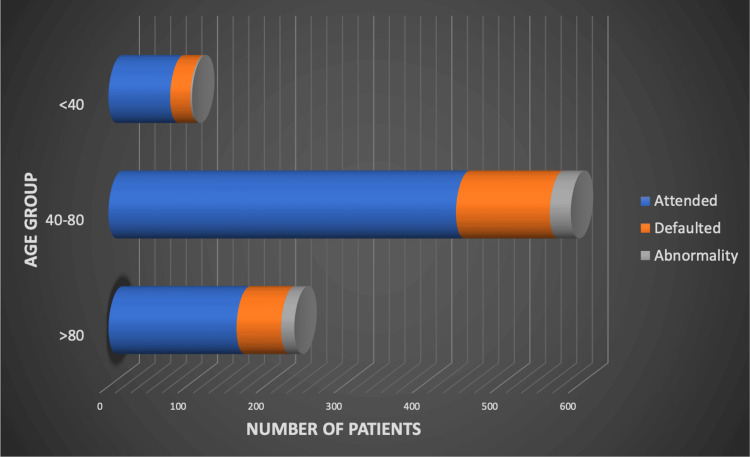
Virtual pneumonia clinic (VPC) referrals by age group, attendance, and abnormalities. Horizontal stacked bars show the distribution of referrals across three age categories (<40, 40–80, >80 years), broken down by follow-up attendance (blue), non-attendance after two invitations (orange), and presence of residual radiographic abnormalities (Grey). The 40–80 age group represented the majority of referrals and accounted for the highest number of abnormalities detected.

Figure 2 illustrates VPC referrals and outcomes by age group.

Radiographic findings

Among the 695 patients who attended follow-up imaging, 46 (6.6%) exhibited persistent radiographic abnormalities. The most frequently observed abnormality was pneumonia-related scarring, identified in 14 patients. Bronchiectasis and pleural effusion were each noted in seven patients, while five patients had evidence of atelectasis. Two patients were found to have pleural plaques, and eight were diagnosed with suspected lung cancer. All suspected malignancies were subsequently confirmed and referred via the two-week wait (2WW) pathway for discussion at the respiratory MDT meeting.

**Figure 3:**
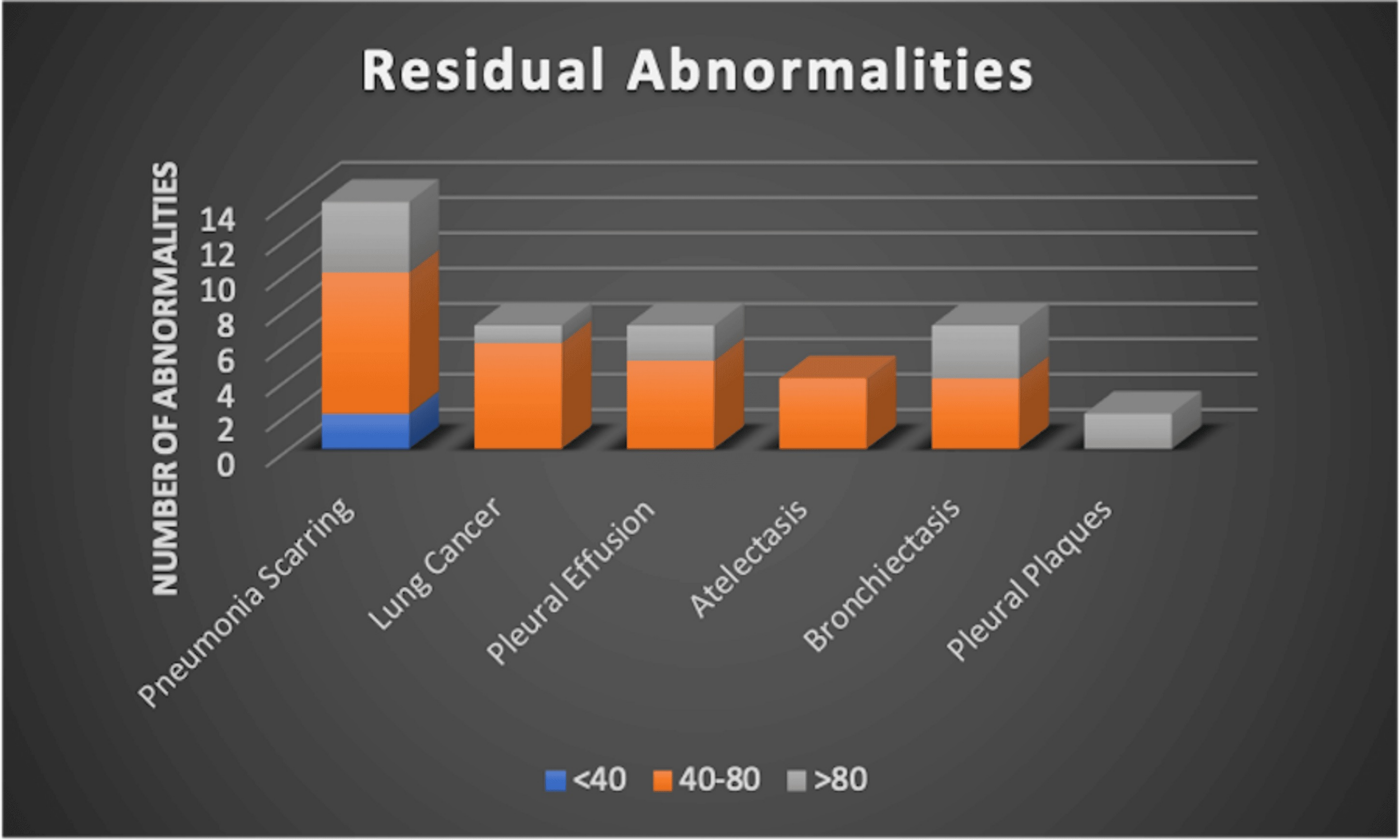
Residual radiographic abnormalities detected at convalescent chest X-ray, stratified by age group. The chart shows the number and age distribution of patients with persistent abnormalities following pneumonia, including scarring, pleural effusion, atelectasis, bronchiectasis, and lung cancer. Most abnormalities were found in patients aged 40 and above, with the highest frequency of scarring in the >80 age group and lung cancer cases primarily in the 40–80 age group.

The distribution of residual abnormalities varied by age group. In patients under 40 years of age, two of 79 (2.5%) had persistent abnormalities. Among those aged 40 to 80 years, 27 of 445 (6.1%) were affected. The highest proportion was observed in patients over 80 years, with 17 of 164 (10.4%) showing residual findings. These age-stratified results are illustrated in Figure 3.

Timeliness of follow-up

The mean time between referral and convalescent CXR was 51.0 days (IQR: 35-63 days). The majority of follow-ups were performed within the BTS-recommended six-week window, although variability existed. These findings suggest that the VPC consistently delivers follow-up imaging within clinically acceptable timeframes, supporting its role in timely post-discharge care.

**Figure 4:**
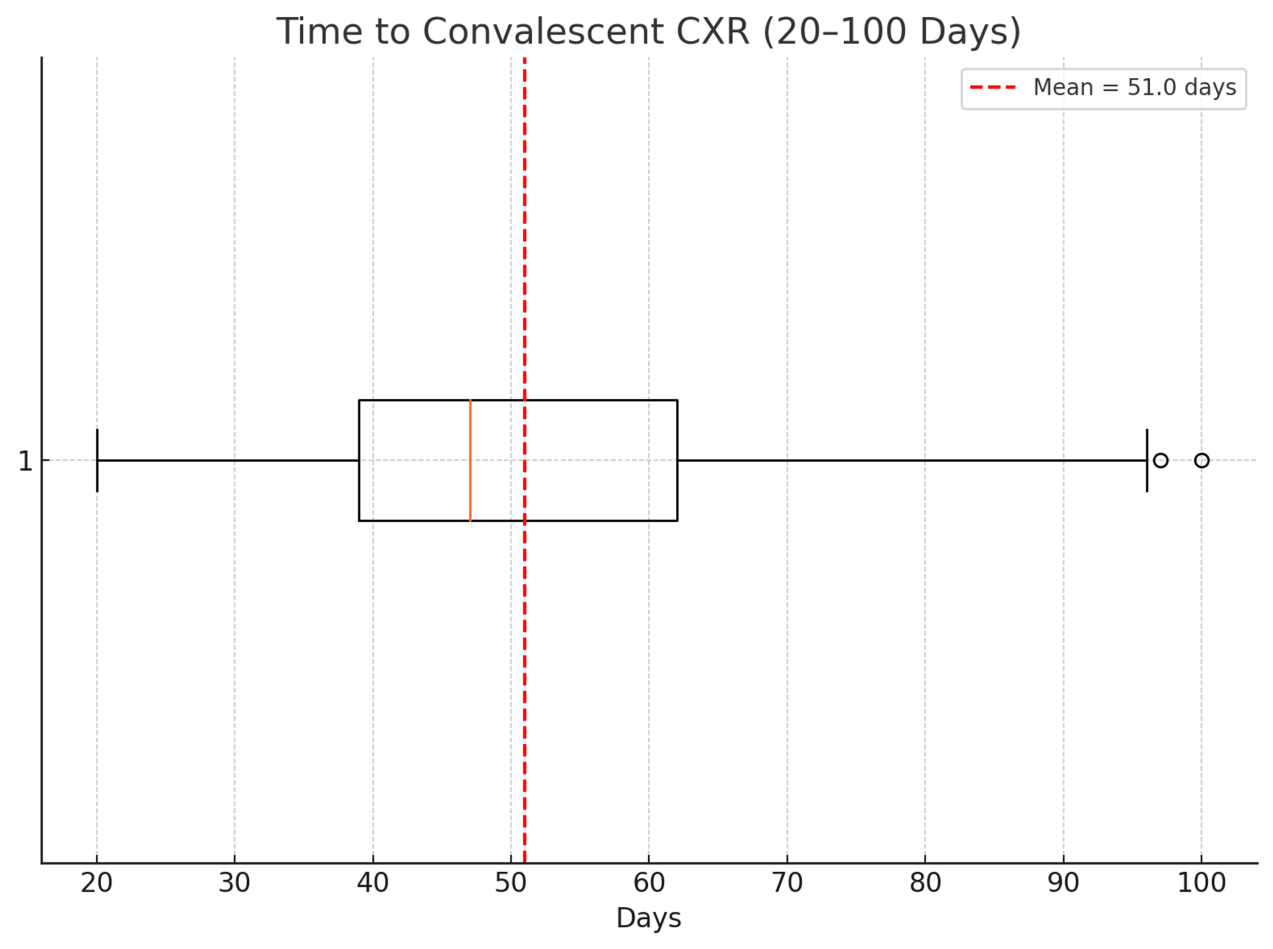
Time to convalescent chest X-ray (CXR) among patients undergoing follow-up. A box-and-whisker plot illustrates the distribution of time intervals between referral and follow-up imaging. The interquartile range spans 35 to 63 days. The mean time to convalescent CXR is indicated by a dashed red line at 51.0 days. Most follow-ups occurred within the British Thoracic Society’s recommended six-week window.

A box-and-whisker plot is presented in Figure 4.

Referrals and service use

**Figure 5:**
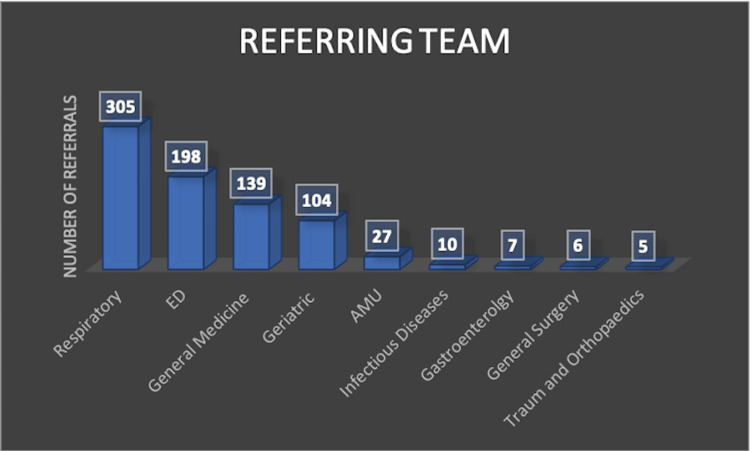
Referring department to the virtual pneumonia clinic (VPC) by volume. The majority of VPC referrals originated from the Respiratory team, followed by the Emergency Department (ED) and General Medicine. Geriatrics, Acute Medical Unit (AMU), and other specialities contributed smaller volumes. Departments with fewer than five referrals were not included in this table. This reflects widespread engagement across inpatient and emergency care streams in identifying patients requiring follow-up.

Most referrals originated from Respiratory (n=305, 34.2%) and Emergency Department (ED) teams (n=198, 22.2%), followed by General Medicine (n=139, 15.6%) and Geriatric Medicine (n=104, 11.6%) (Figure 5). Only 11 referrals (1.2%) were rejected at triage due to adequate prior imaging. Twenty-eight patients (3.1%) died before their scheduled follow-up.

## Discussion

Patient uptake and informal feedback have confirmed that this follow-up service is safe and time-efficient, saving significant numbers of outpatient clinic slots and making it both patient and clinician-friendly. Formal patient feedback is currently being sought retrospectively, but no data is provided in this study. Furthermore, the service is estimated to be cost-effective. We estimate that it saves approximately 90% of the outpatient tariff per patient (equating to £200/€220 per patient) [6]. This conclusion was reached because previously, patients were seen in an outpatient clinic by a respiratory physician six weeks after admission. It meant patients had to travel to the hospital, perhaps on two occasions, for both a CXR and an outpatient appointment. Furthermore, 891 15-minute outpatient appointments are equivalent to 222 hours, while the VPC required only 18 hours of consultant time to review the necessary X-rays, a 92% reduction.

These local findings align with broader evidence across the NHS and international literature. For example, studies have shown that shifting outpatient care to virtual models can generate substantial cost savings. A virtual urology clinic model in the UK demonstrated a 12-month saving of £56,232, alongside increased tariff generation of £72,072 due to improved clinic throughput [7]. Similarly, NHS analysis in the West Midlands projected that converting 10% of outpatient activity to virtual could result in a £5.34 million annual productivity gain [8]. In addition, the Whole System Demonstrator trial from the Department of Health found that tele-health interventions reduced emergency admissions by 20% and hospital bed days by 14% [9].

Beyond direct cost savings, virtual models reduce patient travel time, minimise time off work, and contribute to environmental sustainability. A study reported avoidance of over 4,000 travel miles over four months due to virtual consultations, equating to a potential 12-month reduction of up to four metric tonnes of CO_2_ emissions [7]. These benefits underline the VPC's dual contribution to financial savings and patient-centred, sustainable care.

This service has also helped in the early identification and management of numerous incidental pathologies such as interstitial lung disease and malignancy. It is important to note that patients are evaluated by hospital-based clinicians during their initial admission, and red-flag features suggestive of malignancy or interstitial lung disease (ILD) are typically investigated during this period. The VPC is intended to complement this initial assessment, acting as a post-discharge safety net to detect residual or late-evolving pathology.

It also acts as a valuable educational resource for trainees and students. Medical students and respiratory trainees often sit in to observe these clinical sessions run by a consultant respiratory physician and a respiratory specialist nurse, providing excellent teaching opportunities.

Based on the findings of this review, we have refined our referral criteria to the VPC. As of April 2025, only patients aged over 50 and/or those with ongoing symptoms at discharge, and/or those with recognised respiratory risk factors for malignancy or poor recovery, are invited for a convalescent CXR. This change aims to enhance the efficiency and clinical utility of the service by reducing unnecessary imaging in low-risk populations. A prospective re-audit is currently underway to assess the impact of this change on detection rates, resource use, and patient outcomes.

Limitations

While this study demonstrates the effectiveness of a VPC in identifying radiographic resolution and detecting significant pathology, several limitations may impact the generalisability of our findings.

Firstly, the VPC model was implemented in a large tertiary teaching hospital with access to consultant-led radiograph review and dedicated administrative infrastructure. Institutions without similar resources may find this model more difficult to replicate without adaptation. Additionally, as a single-centre, retrospective review, results may not directly extrapolate to other settings.

Secondly, all patients with a radiological diagnosis of pneumonia were included in the follow-up pathway, regardless of predisposing risk factors or ongoing symptoms, which is not in line with current BTS guidelines. This broad inclusion may limit the specificity of the findings and could lead to unnecessary follow-up imaging in lower-risk individuals. Settings with limited radiology capacity may need a more targeted approach. There may also be a degree of selection bias, as outcomes from non-attending patients (22%) were not available, which could influence estimates of residual pathology prevalence.

Thirdly, while the short-term radiographic and diagnostic outcomes were measured, this study does not provide long-term clinical follow-up data such as mortality, readmission rates, or functional recovery. This limits our ability to evaluate the sustained clinical benefits or potential missed complications of a virtual-only follow-up pathway.

Finally, while informal feedback supports the service’s acceptability, formal patient satisfaction data is not yet available. A prospective evaluation of patient and clinician perspectives would provide a more comprehensive understanding of the service’s utility and sustainability. Further studies in varied healthcare environments with both resource-rich and resource-limited settings, involving multiple centres, are needed to evaluate the scalability and adaptability of the VPC model.

## Conclusions

The VPC remains a highly effective and scalable model for post-pneumonia follow-up, offering timely imaging, early identification of serious pathology, and substantial efficiency gains. Over the past decade, it has evolved into a high-throughput, consultant-led virtual service, with most follow-up chest X-rays occurring within the recommended time frame (mean: 51.0 days; IQR: 35-63). Residual abnormalities were more common in older patients and those with risk factors, informing a recent refinement of referral criteria to target individuals over 50, those with persistent symptoms, or known respiratory risks.

The clinic continues to deliver significant operational benefits, including a 92% reduction in consultant time and an estimated £200 saving per patient. These outcomes align with wider evidence supporting virtual outpatient models as tools to reduce hospital burden, improve clinic throughput, and minimise environmental impact. The VPC has also supported early discharge and acted as a safety net for detecting complications. With referral volumes having tripled since its inception, the service has adapted to demand without compromising safety. This model offers a replicable framework for other NHS trusts and international health systems seeking to optimise outpatient care while maintaining diagnostic quality.

## References

[1] (2025). National Institute for Health and Care Excellence (NICE): Chest infections - adult. https://cks.nice.org.uk/topics/chest-infections-adult/background-information/prevalence/.

[2] Woodhead MA, Macfarlane JT, McCracken JS, Rosec DH, Finch RG (1987). Prospective study of the aetiology and outcome of pneumonia in the community. Lancet.

[3] Macfarlane JT, Finch RG, Ward MJ (1982). Hospital study of adult community-acquired pneumonia. Lancet.

[4] Lim WS, Baudouin SV, George RC (2009). BTS guidelines for the management of community acquired pneumonia in adults: Update 2009. Thorax.

[5] Wilcox CR, Krishnan JV, Duffus C, Marshall BG (2017). Three years of experience with a novel "virtual" pneumonia follow-up clinic. Eur Respir J.

[6] (2024). NHS England: National Cost Collection. https://www.england.nhs.uk/costing-in-the-nhs/national-cost-collection/.

[7] Miah S, Dunford C, Edison M (2019). A prospective clinical, cost and environmental analysis of a clinician-led virtual urology clinic. Ann R Coll Surg Engl.

[8] (2024). The Strategy Unit: The economic impact of outpatient appointments for West Midlands CCGs. November.

[9] (2024). Department of Health: Whole System Demonstrator Programme: Headline findings: December 2011. https://www.gov.uk/government/publications/whole-system-demonstrator-programme-headline-findings-december-2011.

